# Inversion Method of the Young’s Modulus Field and Poisson’s Ratio Field for Rock and Its Test Application

**DOI:** 10.3390/ma15155463

**Published:** 2022-08-08

**Authors:** Yanchun Yin, Guangyan Liu, Tongbin Zhao, Qinwei Ma, Lu Wang, Yubao Zhang

**Affiliations:** 1 School of Aerospace Engineering, Beijing Institute of Technology, Beijing 100081, China; 2 College of Energy and Mining Engineering, Shandong University of Science and Technology, Qingdao 266590, China

**Keywords:** rock, Young’s modulus, Poisson’s ratio, parameters field, inversion method

## Abstract

As one typical heterogeneous material, the heterogeneity of rock micro parameters has an important effect on its macro mechanical behavior. The study of the heterogeneity of micro parameters is more important to reveal the root cause of deformation and failure. However, as a typical heterogeneous material, the current testing and inversion method is not suitable for micro parameters measurement for the rock. Aiming at obtaining the distribution of micro Young’s modulus and micro Poisson’s ratio of the rock, based on the digital image correlation method (DIC) and finite element method (FEM), this paper proposed a parameter field inversion method, namely the DF-PF inversion method. Its inversion accuracy is verified using numerical simulation and laboratory uniaxial compression test. Considering the influences of heterogeneity, stress state and dimension difference, the average inversion error of Young’s modulus field and Poisson’s ratio field are below 10%, and the proportion of elements with an error of less than 15% accounts for more than 86% in the whole specimen model. Compared with the conventional measuring method, the error of macro Young’s modulus and macro Poisson’s ratio calculated by the DF-PF inversion method is less than 2.8% and 9.07%, respectively. Based on the statistical analysis of Young’s modulus field and Poisson’s ratio field, the parameter homogeneity and quantitative function relation between the micro parameter and the principal strain can also be obtained in laboratory tests. The DF-PF inversion method provides a new effective method of testing Young’s modulus field and Poisson’s ratio field of the rocks under complex stress states.

## 1. Introduction

Rock is a typical heterogeneous material. Its microstructure and micro parameter distribution are the main controlling factors leading to strain localization, stress concentration, nonlinear damage evolution, and so on [1,2,3,4]. The study of rock heterogeneity is very important for revealing the mechanism of rock deformation and failure and even guiding the stability control of rock mass engineering. Therefore, in the field of rock mechanics, the microstructure characterization and micromechanical properties research of rocks have always been a hot issue.

At present, rock heterogeneity is mainly studied by numerical simulation methods. Weibull function [5,6] and digital image processing technology (DIP) [7,8] are often used to characterize the heterogeneity of rock microparameters and microstructures. By using the Weibull function, Chen et al. [9] and Pan et al. [10] studied the effect of rock parameter homogeneity on its macro mechanical behavior. Based on the micrographs of rocks, Shah et al. [11] built a microstructure-based numerical model and evaluated the microscale failure response of various weathering grade sandstones. These studies have a certain significance for revealing the mechanical properties of the rock. However, in these kinds of numerical simulation methods, the microparameters are hard to quantitatively assigned. Even if it is characterized by Weibull function, it is based on artificial assumptions, and is inconsistent with the real distribution of the rock. Therefore, the accurate assignment of micro parameters in microstructure numerical models is important to research.

The laboratory test is another commonly used method to study rock microproperties. The commonly used test methods include computerized tomography (CT) [12], scanning electron microscope (SEM) [13], digital image correlation method (DIC) [14,15,16,17], and others. CT and SEM are always used to obtain the microstructures and microfractures distribution of the rock. Using DIC, the rock’s deformation field and strain localization characteristics can be analyzed. The laboratory test methods mainly focus on the research of rock microstructures and lack effective micro parameters testing methods.

Combining numerical simulation methods and laboratory test methods, a variety of parameter inversion methods for materials have been proposed, such as the virtual field method [18] and finite element model updating method (FEMU) [19]. These kinds of methods can obtain material mechanical parameters under complex stress. By using weight FEMU and DIC, Mathieu et al. [20] estimated the parameters of the pure titanium sample under tensile loading. Ogierman et al. [21] proposed a novel two-step optimization procedure inversion method of the elastic properties of the composite constituents, which improved the solution efficiency. Based on the digital speckle correlation method (DSCM) and finite element method (FEM), Song et al. [22,23] proposed the DSCM-FEM inversion method, which can obtain the Young’s modulus and Poisson’s ratio of rock soil materials.

The above inversion methods can obtain the macro Young’s modulus and other material parameters under a certain stress state, which provide ideas for measuring microparameters. Liu et al. [24,25] proposed a double iterative inversion method based on DIC and FEM. They obtained Young’s modulus field (i.e., elements micro Young’s modulus distribution on specimen surface) and damage variable field of graphite material. In this method, it is assumed that the damage variable of the micro Young’s modulus of the whole model conforms to the same equation, which is suitable for the inversion of Young’s modulus field of homogeneous materials such as graphite. However, for rock materials affected by the diagenetic process, in-situ stress environment, engineering construction disturbance and specimen processing, the rock before the test has certain initial damage, and the microparameters and macroparameters have obvious discreteness [26,27,28]. The parameter damage of elements of the specimen is difficult to be described by the same quantitative equation. It is verified that this method cannot obtain the Young’s modulus field of heterogeneous rock.

As a typical heterogeneous material, the current parameter inversion method is not suitable for micro parameters inversion for the rock. In order to effectively obtain the micro parameters of the rock, this paper proposed a parameter field inversion method for rocks based on DIC and FEM, namely DF-PF inversion method. This method can perform simultaneous inversion of Young’s modulus field and Poisson’s ratio field (elements’ micro Poisson’s ratio distribution on specimen surface). The accuracy of the inversion results is verified by a numerical simulation test, and the inversion test of laboratory rock uniaxial compression test was carried out. The tests reveal that the DF-PF inversion method can obtain Young’s modulus field and Poisson’s ratio field of rocks under a complex stress state.

## 2. DF-PF Inversion Method

One key link in the parameter inversion process based on DIC and FEM is the matching between experimental and simulated strain fields. The strain field of rock is not only related to Young’s modulus, but Poisson’s ratio is also an important factor, and micro Poisson’s ratio of elements is also heterogeneous. Therefore, to ensure the accuracy of inversion results, Poisson’s ratio field should be carried out simultaneously during the inversion of rock Young’s modulus field.

**Figure 1:**
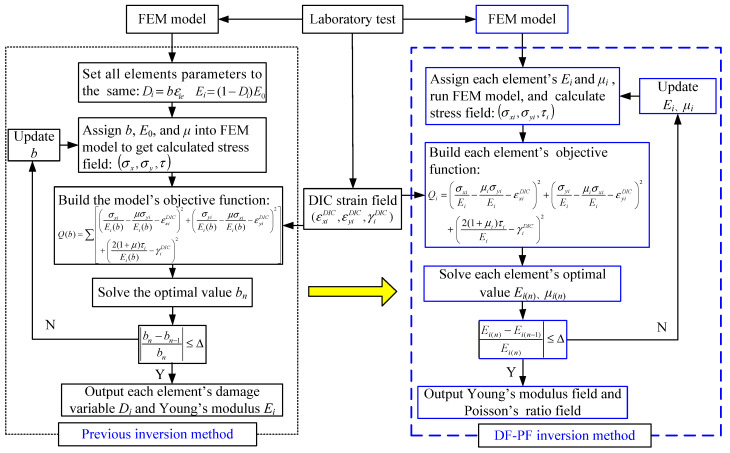
Flow charts of the DF-PF inversion method.

Based on the high-efficiency double iterative inversion method proposed by the authors’ previous work [24], this paper optimizes its objective function and iterative process. It puts forward the DF-PF inversion method, which can realize the synchronous inversion of rock Young’s modulus field and Poisson’s ratio field, as shown in Figure 1. The specific methods are as follows:

(1) Carry out the laboratory test of rock specimen, and obtain the real strain field on the specimen surface by DIC method.
(2) Then, FEM is used to establish the numerical model, and the geometric dimensions and boundary conditions of the numerical model are consistent with those in the laboratory test. Assign each element’s micro Young’s modulus *E_i_* and micro Poisson’s ratio *μ_i_* separately, and their initial value can be set as the results measured by ISRM suggested method.
(3) Export the stress field obtained by FEM, and calculate the strain field by Hooke’s law. Then, an objective function *Q_i_* is established as the squared difference between the strains measured with the DIC and the strains calculated by FEM. Each element *i* establishes an independent objective function *Q_i_*, in which micro Young’s modulus *E_i_* and micro Poisson’s ratio *μ_i_* are taken as the inversion variables. The idea is to iteratively minimize the objective function with respect to Young’s modulus *E_i_* and micro Poisson’s ratio *μ_i_*. The objective function *Q_i_* is:
  (1) $$Q_{i}={\left(\frac{\sigma_{xi}}{E_{i}}-\frac{\mu_{i}\sigma_{yi}}{E_{i}}-\varepsilon_{xi}^{DIC}\right)}^{2}+{\left(\frac{\sigma_{yi}}{E_{i}}-\frac{\mu_{i}\sigma_{xi}}{E_{i}}-\varepsilon_{yi}^{DIC}\right)}^{2}\;+{\left(\frac{2(1+\mu_{i})\tau_{i}}{E_{i}}-\gamma_{i}^{DIC}\right)}^{2}$$
  where *σ_xi_*, *σ_yi_*, *τ_i_* is the horizontal stress, vertical stress and shear stress of element *i* obtained by FEM, and $\varepsilon_{xi}^{DIC}$, $\varepsilon_{yi}^{DIC}$, $\gamma_{i}^{DIC}$ is the horizontal strain, vertical strain and shear strain of element *i* obtained by the DIC test.

To minimize the objective function *Q_i_* by iteratively seeking for optimal parameters value, the Nelder–Mead simplex method is used [24]. For an optimization problem with two parameters, the Nelder–Mead simplex method is a classical and successful optimization method for unconstrained optimization problems without requiring gradient information.

(4) Solve the objective function *Q_i_* with the Nelder–Mead simplex method, and the optimal micro Young’s modulus *E_i_*_(*n*)_ and micro Poisson’s ratio *μ_i_*_(*n*)_ of each element in the current iterative step *n* can be obtained.
(5) Input the new micro Young’s modulus and micro Poisson’s ratio of each element into the FEM model and repeat steps (2), (3) and (4). When the difference in the value of each element’s micro Young’s modulus between the two iteration steps is less than the allowable error Δ, stop the iteration. Then output the micro Young’s modulus and micro Poisson’s ratio of each element, and draw Young’s modulus field and Poisson’s ratio field.

In the DF-PF inversion method, the damage variable *D* is removed, and Young’s modulus field and Poisson’s ratio field obtained by inversion are the results considering damage evolution. Thus, this method can be used for micromechanical parameter inversion of the rock both in elastic and plastic deformation states.

## 3. Verification of the Method

### 3.1. Verification Thought

**Figure 2:**
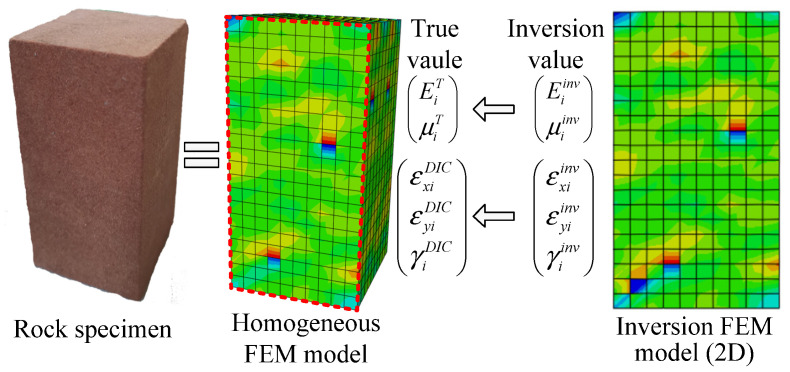
Verification thought of the inversion method.

Due to the obvious discreteness of rocks, the distribution of micro Young’s modulus and micro Poisson’s rock ratio cannot be determined quantitatively in laboratory tests. It is difficult to verify the accuracy of the DF-PF inversion method by laboratory test method. Therefore, this paper verifies the inversion method using the numerical simulation method. Firstly, establish one heterogeneous rock specimen model through Abaqus, and take the strain field on one surface as DIC strain field data, which surrogate the laboratory test results. Thus, the micro parameters of the rock are known. Then, the DF-PF inversion method is used to inverse Young’s modulus field and Poisson’s ratio field on the rock surface. Finally, the true value of micro parameters of the specimen is compared with the inversion results to verify the accuracy of the results. The specific method is shown in Figure 2.

In the rock specimen model simulating the laboratory test, micro Young’s modulus and micro Poisson’s ratio of elements of the rock model were heterogeneous, and its homogeneity was set by the shape parameter *m* in the Weibull function [9,10]. The greater the homogeneity *m* is, the more homogeneous the micromechanical parameters of rock are. Meanwhile, the damage variable *D* was added to the model, which is related to the strain of the element [29]. The micro Young’s modulus and micro Poisson’s ratio of each element was changed with increasing the strain, as shown in Equation (2). Parameters’ heterogeneous distribution and damage evolution were realized by writing a user material subroutine (UMAT).

(2) $$\{\begin{array}{c} E_{i}=E_{i0}(1-D)\\ \mu_{i}=\mu_{i0}(1-D)\\ D=ae^{b\varepsilon_{e}}+c \end{array}$$

where *E_i_*_0_ and *μ_i_*_0_ are the initial value of micro Young’s modulus and micro Poisson’s ratio of element *i*; *ε_e_* is the equivalent strain; *a*, *b*, and *c* is the constant.

### 3.2. Verification Schemes

In this section, five factors influencing the inversion accuracy were studied, which were micro Young’s modulus homogeneity *m_E_*, micro Poisson’s ratio *m_μ_*, stress level *σ*, stress state and model dimension difference (i.e., the laboratory test is a 3D-dimensional model, and the inversion adopts a 2D-dimensional model). Five verification schemes were mainly designed, as shown in Table 1.

(1) In scheme I, a 2D uniaxial compression test was carried out to study the influences of micro Young’s modulus homogeneity and stress level. The size of the rock specimen model was 50 mm × 100 mm and was divided into 800 quadrilateral elements. The initial macro Young’s modulus and macro Poisson’s ratio were set to 10 GPa and 0.25. Micro Young’s modulus was set as heterogeneity, while micro Poisson’s ratio was homogeneous.
(2) In scheme II, the 2D diametral compression test of the ring specimen was carried out to study the influence of the stress state. Compared with the uniaxial compression test, the element is in a complex stress state in the diametral compression test. The ring’s outer and inner diameters were 100 mm and 60 mm, respectively, divided into 608 quadrilateral elements.
(3) Scheme III mainly studied the influence of the dimensional difference between the test and inversion models on the inversion results. The test model was a 3D uniaxial compression specimen, a cuboid of 50 mm × 50 mm × 100 mm and divided into 16,000 hexahedral elements. The inversion model was still the 2D uniaxial compression specimen, the same as in scheme I.
(4) Scheme IV mainly studied the inversion accuracy when micro Poisson’s ratio and micro Young’s modulus were both heterogeneous, and it was researched by 2D uniaxial compression test and 3D uniaxial compression test.

In the verification process, the following indexes were mainly used to evaluate the accuracy of the results:

(1) *e_εx_*, *e_εy_*, and *e_γ_*: the mean value of the relative error of horizontal strain, vertical strain and shear strain between the DIC strain field and inversion strain field.
(2) *R_εx_*, *R_εy_*, and *R_γ_*: the correlation coefficient of horizontal, vertical, and shear strain between DIC strain field and inversion strain field. The more the correlation coefficient tends to 1, the better the correlation.
(3) *e_E_*, and *S_E_*: the relative error and its standard deviation between the true value and the inversion value of Young’s modulus field.
(4) *e_μ_*, and *S_μ_*: the relative error and its standard deviation between the true value and the inversion value of Poisson’s ratio field.
(5) *R_i_*: the proportion of the elements in the whole specimen.

### 3.3. Results and Analysis

**Figure 3:**
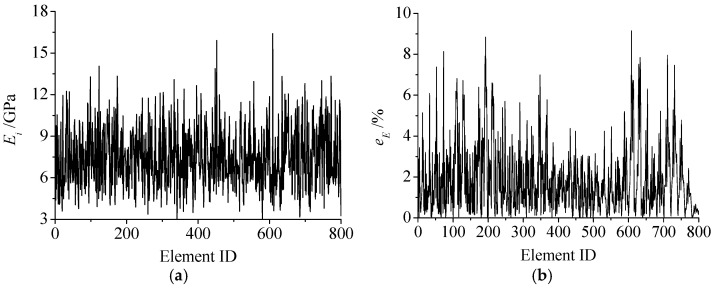
(**a**) Inversion value and (**b**) relative error of micro Young’s modulus of the specimen with homogeneity *m_E_* = 2.

The results of the four schemes show that the correlation coefficient between the DIC strain field and the inversion strain field of all tests was greater than 0.93, and the relative error was less than 2%. The strain data almost completely coincide, which shows high inversion accuracy of the strain field. The inversion results of the specimen with homogeneity *m_E_* = 2 and an axial stress *σ =* 15 MPa in scheme I were taken as an example. Micro Young’s modulus inversion results are shown in Figure 3. The maximum relative error *e_E_* of elements’ micro Young’s modulus between the true value and the inversion value is less than 10%, the mean value is 1.89%, and the standard deviation *S_μ_* is 1.68%.

**Figure 4:**
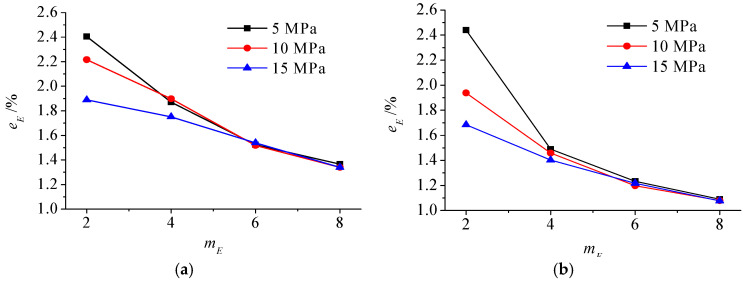
(**a**) The mean value and (**b**) standard deviation of relative error of Young’s modulus field of Scheme I.

The inversion error of Young’s modulus field of scheme I is shown in Figure 4. The stress level only significantly impacts the inversion results of rocks with small homogeneity (such as 2 and 4). With the decrease in homogeneity, the mean value and standard deviation of the relative error of the inversion results gradually increase. However, the mean value is still less than 2.5%, which shows that the inversion accuracy meets the requirements.

**Figure 5:**
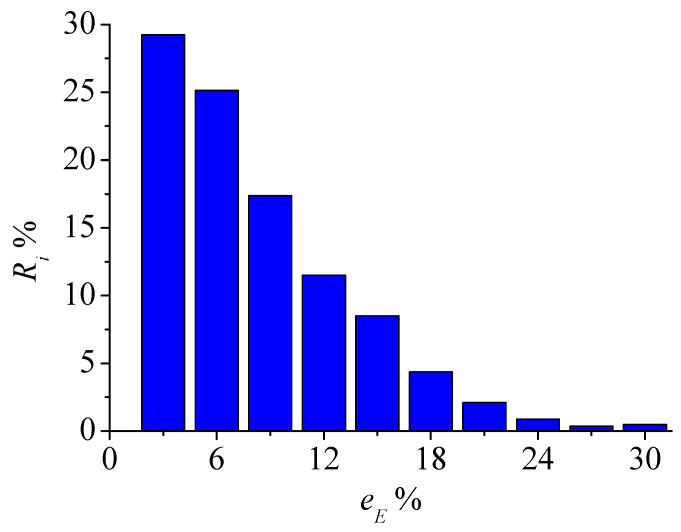
Statistics of the inversion error of Young’s modulus field of scheme III.

Young’s modulus field inversion results of schemes II and III were listed in Table 2. The mean value and standard deviation of the relative error of scheme II are relatively small, indicating that the complexity of the specimen stress state has little impact on the inversion accuracy of micro Young’s modulus. The relative error of scheme III is larger than that of scheme II. For example, when the homogeneity *m_E_* = 2, the average relative error is 6.61%, but the micro Young’s modulus error of most elements is still small, and the proportion *R_i_* of elements with an error less than 15% is 91.75%, as shown in Figure 5. Considering the dimensional difference between the laboratory test and the inversion model, the deformation field of the outer surface of the rock specimen is not only related to the elements on the surface. It is also affected by the associated internal elements, resulting in a larger inversion error of micro Young’s modulus of some elements. However, this part accounts for a small proportion, and the inversion accuracy of Young’s modulus field of the specimen can still meet the test requirements.

**Figure 6:**
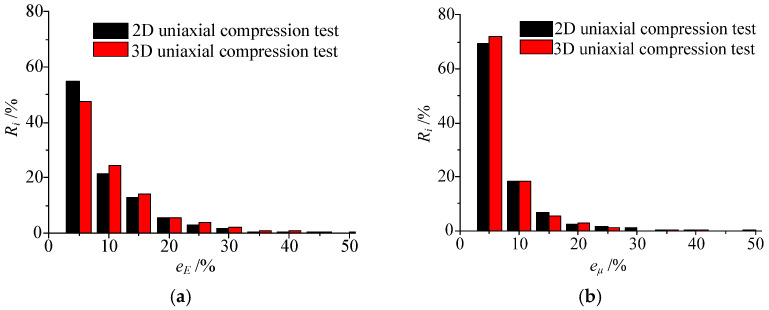
Statistics of the inversion error of (**a**) Young’s modulus field and (**b**) Poisson’s ratio field when Poisson’s ratio is heterogeneous.

When considering Poisson’s ratio heterogeneity *m_μ_*, the inversion results of Young’s modulus field and Poisson’s ratio field are listed in Table 3. Compared with the homogeneous Poisson’s ratio, its heterogeneous distribution has a certain impact on the inversion result. The maximum value of the relative error of Young’s modulus field is 9.89%, while the error of most elements is still small. The proportion of elements with an error of less than 10% accounts for more than 72%, and the proportion of elements with an error of less than 15% accounts for more than 86%, as shown in Figure 6a. For Poisson’s ratio field, the average relative error of the 2D uniaxial compression test is 6.18%, the proportion of elements with error less than 10% is more than 87%, and the proportion of elements with error less than 15% is more than 94%, as shown in Figure 6b.

## 4. Application in Laboratory Test

### 4.1. Test Scheme

**Figure 7:**
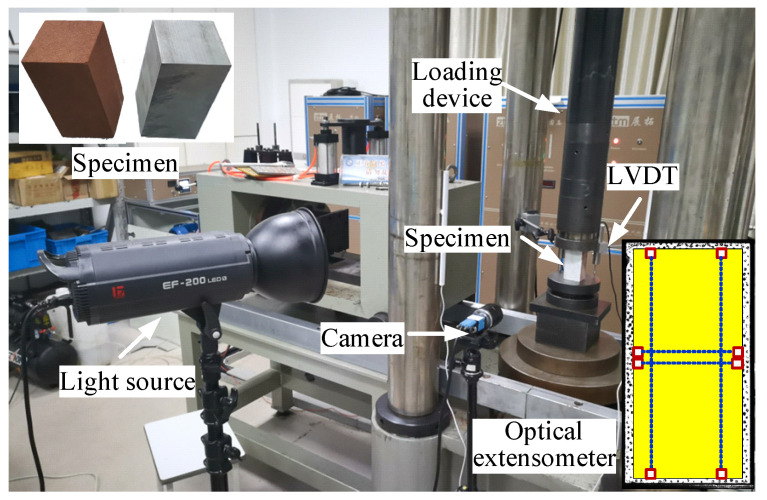
Testing system.

In order to verify the effectiveness of the DF-PF inversion method in a laboratory test, aluminum and sandstone specimens were selected for the uniaxial compression test, and the specimen was cuboids of 50 mm × 50 mm × 100 mm. The loading device was RLJW-2000 rock mechanics testing machine, developed by Shandong University of Science and Technology (Qingdao, China). The loading speed was 0.1 mm/min. During the test, the axial deformation of the specimen was monitored by using LVDT displacement sensor, produced by Changchun Testing Machine Co. Ltd (Changchun, China). DIC test was carried out simultaneously, and four groups of optical extensometers were arranged [17]. The sampling frequency of the speckle image was 10 frames/s. The specific test system is shown in Figure 7.

### 4.2. Inversion Results

**Figure 8:**
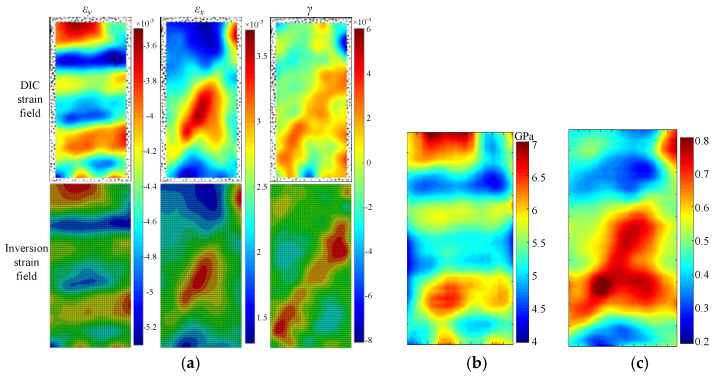
(**a**) Strain field; (**b**) Young’s modulus field and (**c**) Poisson field of the sandstone under axial stress of 26 MPa.

Since the inversion results of the strain field, Young’s modulus field and Poisson’s ratio field of aluminum and sandstone are similar, the sandstone with low homogeneity was analyzed as an example. The inversion results of the sandstone parameter field under axial stress of 26 MPa (99%*σ_c_*) are shown in Figure 8.

For axial strain *ε_y_* field and transverse strain *ε_x_* field, the average relative error between the test results and the inversion results is less than 2%, and the correlation coefficient is greater than 0.99. However, the inversion error of the shear strain *γ* field is large. Its correlation coefficient is 0.537, and the mean value of the relative error is 49.37%. However, its absolute error is small, and the mean value is only 1.47 × 10^−6^. Since the mean value of shear strain field *γ* is 2.97 × 10^−6^, its relative error is relatively larger. Overall, the average inversion error of the three strain fields is 17.13%, and the correlation coefficient is 0.842. The strain localization region in the DIC results and the inversion results are similar, as shown in Figure 8a. DIC strain field matches well with inversion strain field for rocks with large discreteness.

Young’s modulus and Poisson’s ratio fields show obvious localization characteristics. In the uniaxial compression test, the axial strain is related to Young’s modulus, and the transverse strain is related to Poisson’s ratio. The inversion results show that the distribution patterns of Young’s modulus field and axial strain *ε_y_* field, Poisson’s ratio field and transverse strain *ε_x_* field are similar, as shown in Figure 8b,c. But they are not exactly the same due to rock heterogeneity. At the same time, affected by the loading end effect, the Poisson’s ratio in the middle of the specimen is significantly greater than that in the upper and lower ends.

**Figure 9:**
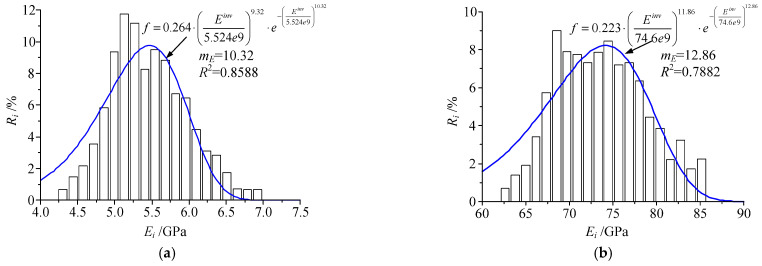
Statistical distribution of micro Young’s modulus of (**a**) sandstone and (**b**) aluminum.

The statistical distribution of micro Young’s modulus of sandstone and aluminum is shown in Figure 9. The distribution form of micro Young’s modulus is well fitted with the Weibull function [10], and the homogeneity of aluminum (*m_E_* = 12.86) is greater than that of sandstone (*m_E_* = 10.32). The Poisson’s ratio also presents a similar law.

The above analysis shows that the inversion results of Young’s modulus field and Poisson’s ratio field obtained by the DF-PF inversion method are reasonable and reliable in the laboratory.

### 4.3. Comparison with Conventional Test

Based on Young’s modulus field and Poisson’s ratio field obtained by the DF-PF inversion method, the mean value of the whole field or local field data can be used to calculate the macro Young’s modulus and Poisson’s ratio of the specimen. Therefore, the accuracy of the inversion method can be further verified by comparing the macro parameters calculated by the DF-PF inversion method with the conventional test results. In the laboratory test, the macro Poisson’s ratio was measured and calculated by DIC optical extensometer [17], and the macro Young’s modulus was measured by a displacement sensor and DIC optical extensometer. In the DF-PF inversion method, the mean value of the whole field data of Young’s modulus field was taken as the macro Young’s modulus. The mean value of the Poisson’s ratio field data in the area surrounded by the two groups of transverse DIC optical extensometers (as shown in Figure 7) was taken as the macro Poisson’s ratio. The comparison and relative error of macro mechanical parameters obtained by the three methods are listed in Table 4. For Poisson’s ratio, with the increase in stress level, the Poisson ratio increases gradually. The Poisson’s ratio of sandstone under axial stress of 99%*σ_c_* exceeds 0.5, indicating that the sandstone has already entered the plastic deformation stage, which is consistent with the research conclusions of the literature [30,31].

Compared with the results of the laboratory sensor method and DIC optical extensometer method, the average error of macro Young’s modulus obtained by the DF-PF inversion method is less than 2.5%. The average error of macro Poisson’s ratio is less than 9.07%, which can meet the accuracy requirements of rock mechanical parameters with large discreteness. At the same time, the error of mechanical parameters of aluminum is less than that of sandstone, which is the same as the simulation test conclusion. That is, the more homogeneous the specimen material is, the smaller the inversion error is.

### 4.4. Micro Parameter Evolution Analysis

**Figure 10:**
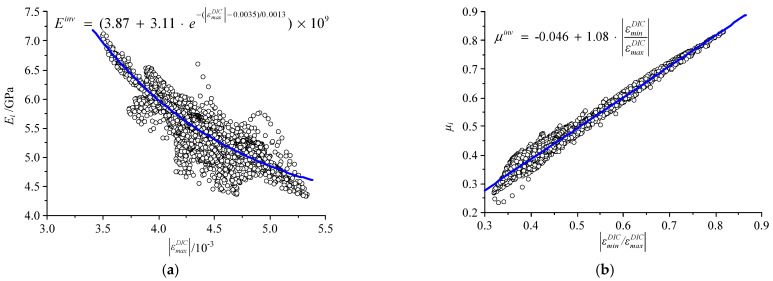
Evolution curves of (**a**) micro Young’s modulus and (**b**) Poisson ratio.

In the construction of a rock constitutive model, the evolution equation of Young’s modulus is the key to the consistency between the constitutive model and test. It is often determined by function fitting with a stress-strain curve combined with a theoretical model. In the DF-PF inversion method, the values of micro Young’s modulus and micro Poisson’s ratio under different DIC strains of the elements were obtained, and the function fitting of the evolution equations of Young’s modulus and Poisson’s ratio were also carried out. It is verified that the micro Young’s modulus and the maximum principal strain $\left|\varepsilon_{max}^{DIC}\right|$ fit well, and the relationship between them is an exponential function, which is consistent with the heterogeneous damage constitutive model of rock [32]. The fitting between micro Poisson’s ratio and principal strain ratio $\left|\varepsilon_{min}^{DIC}/\varepsilon_{max}^{DIC}\right|$ is good, and they have a linear relationship, as shown in Figure 10.

## 5. Discussion

For typical heterogeneous materials like rocks, the mechanical parameters are very discrete. Previous works showed that the 95% confidence interval for rock mechanical parameters is (1 ± 20%) or higher [33,34]. For the DF-PF inversion method, the average error of Young’s modulus field and Poisson’s ratio field is below 10% in the numerical simulation test. The error of macro parameters is less than 9.07% in a laboratory test. It is verified that the DF-PF inversion method can meet the application requirements of rock parameter field inversion.

Double iterative inversion method [24] is a high-efficiency Young’s modulus field and damage field inversion method. But this method is only suitable for homogeneous materials, not for rocks. Compared with the double iterative inversion method, the DF-PF inversion method is an effective inversion method for rocks while retaining high computational efficiency. Since this method is proposed based on DIC and FEM, the DF-PF inversion method is suitable for rocks in elastic and plastic deformation stages. This method will not be applicable when the rock generates obvious macro fractures.

Further, the DF-PF method can be used with the microstructure simulation method. In the microstructure simulation model, the size and distribution of mineral structures can be consistent with real rocks based on DIP [1,7,8]. But the micro parameters of different minerals are hard to assign accurately, and a trial and error method is generally used to determine the appropriate parameter values. For this problem, the DF-PF inversion method can provide accurate micro parameters for the microstructure simulation model.

## 6. Conclusions

In order to obtain the distribution of micro Young’s modulus and micro Poisson’s ratio of the rock, based on DIC and FEM, a parameters field inversion method named DF-PF inversion method is proposed, and its inversion accuracy is verified. The main outcome of the study can be summarized as follows:

(1) DF-PF inversion method provides a new effective method for the inversion of Young’s modulus field and Poisson’s ratio field for rocks in elastic and plastic deformation stages without obvious macro fractures. The average relative error is less than 10%.
(2) The Young’s modulus field and Poisson’s ratio field are obtained in the laboratory test. The parameters field shows obvious localization characteristics, which provides abundant data for the research of the nonlinear damage evolution of rocks under different stress states.
(3) Compared with the conventional measuring method, the error of macro Young’s modulus and macro Poisson’s ratio calculated by the DF-PF inversion method is less than 2.8% and 9.07%, respectively. The DF-PF inversion method can be used to measure rock macro mechanical parameters.
(4) The relationship between micro Young’s modulus and maximum principal strain is an exponential function, and the relationship between micro Poisson’s ratio and principal strain ratio is linear. It provides a new method for the determination of damage equation in the rock heterogeneous damage constitutive model.

## Figures and Tables

**Table 1:** Verification schemes of the DF-PF inversion method.

Scheme	Influence Factor	Test Type	*m*	*σ*/MPa
I	*m_E_* and *σ*	2D uniaxial compression test	2, 4, 6, 8	5, 10, 15
II	Stress state	2D diametral compression test	2, 4, 6	10
III	Model dimensional difference	3D uniaxial compression test	2, 4, 6	10
IV	*m_E,_* and *m_μ_*	2D and 3D uniaxial compression test	2	10

**Table 2:** Inversion error of Young’s modulus field of schemes II and III.

Influence Factor	*e_E_*			*S_E_*		
*m_E_*	2	4	6	2	4	6
Scheme II	1.87%	0.88%	0.82%	1.80%	1.23%	0.92%
Scheme III	6.61%	3.47%	2.60%	5.25%	2.97%	2.21%

**Table 3:** Inversion error of Young’s modulus field and Poisson ratio field of Scheme IV.

Test Model	*e_E_*	*e_μ_*
2D uniaxial compression test	8.67%	6.18%
3D uniaxial compression test	9.89%	5.83%

**Table 4:** Comparison of the inversion results and the conventional testing results.

Specimen	Stress/MPa	Macro Young’s Modulus/GPa			Macro Poisson’s Ratio	
		DF-PF	LVDT (Error)	DIC (Error)	DF-PF	DIC (Error)
Sandstone	18(69%*σ_c_*)	6.16	5.89 (4.58%)	5.93 (3.88%)	0.314	0.344 (8.72%)
	22(84%*σ_c_*)	5.85	6.09 (3.94%)	5.88 (0.51%)	0.436	0.463 (5.83%)
	26(99%*σ_c_*)	5.52	6.00 (8.00%)	5.45 (1.28%)	0.604	0.681 (11.3%)
	Mean value	5.84	5.99 (2.50%)	5.75 (1.57%)	0.451	0.496 (9.07%)
Aluminum	160	71.74	71.54 (0.28%)	70.59 (1.63%)	0.139	0.152 (8.55%)
	180	71.49	72.55 (1.46%)	70.00 (2.12%)	0.154	0.167 (7.78%)
	200	74.60	73.53 (1.46%)	72.73 (2.57%)	0.193	0.210 (8.10%)
	Mean value	72.61	72.54 (0.10%)	71.11 (2.11%)	0.162	0.176 (7.96%)

## Data Availability

The data used to support the findings of this study are available from the corresponding authors upon request.
